# Review of eating disorders and oxytocin receptor polymorphisms

**DOI:** 10.1186/s40337-021-00438-0

**Published:** 2021-07-13

**Authors:** Victoria Burmester, Dasha Nicholls, Alexis Buckle, Boban Stanojevic, Marta Crous-Bou

**Affiliations:** 1 Department of Brain Sciences, Division of Psychiatry, Imperial College London, Burlington Danes, The Hammersmith Hospital, Du Cane Road, London, W12 0NN UK; 2 Comprehensive Cancer Centre, Faculty of Life Sciences & Medicine, King’s College London, Rayne Institute, 111 Coldharbour Ln, London, SE5 9RR UK; 3 Laboratory for Radiobiology and Molecular Genetics, “Vinca” Institute of Nuclear Sciences, University of Belgrade, Belgrade, Serbia; 4 Unit of Nutrition and Cancer, Cancer Epidemiology Research Program, Catalan Institute of Oncology (ICO) - Bellvitge Biomedical Research Institute (IDIBELL). L’Hospitalet de Llobregat, Barcelona, Spain; 5 Department of Epidemiology, Harvard T.H. Chan School of Public Health, Boston, MA USA

## Abstract

**Background and aims:**

Oxytocin, a nine amino acid peptide synthesised in the hypothalamus, has been widely recognised for its role in anxiolysis, bonding, sociality, and appetite. It binds to the oxytocin receptor (OXTR)—a G-protein coupled receptor—that is stimulated by the actions of oestrogen both peripherally and centrally. Studies have implicated *OXTR* genotypes in conferring either a risk or protective effect in autism, schizophrenia, and eating disorders (ED). There are numerous DNA variations of this receptor, with the most common DNA variation being in the form of the single nucleotide polymorphisms (SNPs). Two *OXTR* SNPs have been most studied in relation to ED: rs53576 and rs2254298. Each SNP has the same allelic variant that produces genotypes AA, AG, and GG. In this critical review we will evaluate the putative role of rs53576 and rs2254298 SNPs in ED. Additionally, this narrative review will consider the role of gene-environment interactions in the development of ED pathology.

**Findings:**

The *OXTR* SNPs rs53576 and rs2254298 show independent associations between the A allele and restrictive eating behaviours. Conversely, the G allele of the *OXTR* rs53576 SNP is associated with binging behaviours, findings that were also evident in neuroanatomy. One study found the A allele of both *OXTR* SNPs to confer risk for more severe ED symptomatology while the G allele conferred some protective effect. An interaction between poor maternal care and rs2254298 AG/AA genotype conferred increased risk for binge eating and purging in women.

**Conclusions:**

Individual OXTR SNP are unlikely in themselves to explain complex eating disorders but may affect the expression of and/or effectiveness of the OXTR. A growing body of G x E work is indicating that rs53576G homozygosity becomes disadvantageous for later mental health under early adverse conditions but further research to extend these findings to eating pathology is needed. The GWAS approach would benefit this area of knowledge.

## Plain English summary

Oxytocin is a chemical made in the brain that affects human behaviour in areas from anxiety, bonding right through to appetite. Oxytocin works by binding to a specific cellular receptor. In humans, the genes that specify this receptor are found in slightly different versions that are inherited from each parent. Research has suggested that individuals who possess speicfic combinations of oxytocin receptor gene variants may be more susceptible to certain kinds of mental illness. This paper considers two different versions of the oxytocin receptor gene most studied in relation to eating disorders. The two different versions considered in this review do not seem to affect the structure of the oxytocin receptor itself. Together, research indicates that the presence or absence of a particular receptor gene variant in an individual might have some predictive capability in respect of potential susceptibility to eating disorders. However, further research is necessary as some of the findings are contradictory. In addition, environmental factors—such as poor maternal care early in life—have also been demonstrated to be important in determining whether an individual will develop an eating disorder. Research in this area would benefit from non-hypothesis driven studies.

## Background

### Overview of oxytocin

The nine-amino acid peptide oxytocin is synthesised mainly in the hypothalamus and secreted directly within the cerebrum but also released via the posterior pituitary gland to regulate a range of physiologic and metabolic processes [1]. Centrally, oxytocin has a wide distribution and is recognised for its context-dependent behavioural effects in mammals including anxiolysis, bonding, appetite, and sociality [2].

Oxytocin is an anorectic with multifarious hypophagic controls, including homeostatic satiety-related signalling between the peripheral and central nervous system, metabolic modulation, supraoptic nuclei metabolic feedback, and the reduction of feeding reward [3]. Blockade of OXTR in mice results in hyperphagia [4]. Exogenous oxytocin is an effective anorexiant in animals, both via systemic administration [5–7] and intracerebroventricular (ICV) routes [8], which is reversed by antagonists [9–11]. In men [12] and women [13], intranasal oxytocin inhibits postprandial snack intake.

The anorectic effects of chronic oxytocin administration have not been widely studied. However, repeated ICV injections of oxytocin agonist (e-L-β-MePhe^2^) OT, inhibited feeding in rats but also produced tolerance [14]. In diet-induced obese rhesus monkeys, chronic oxytocin across a four-week period effectively reduced body mass [15]. However, chronic oxytocin in mice facilitated observational fear and downregulated oxytocin receptors in the amygdala without tolerance or reductions in body mass, suggesting that oxytocin does not cause organisms to lose weight beyond their limits [16]. In obese humans, chronic oxytocin over an eight-week period reversed obesity and prediabetic changes [17].

Studies in people with Prader Willi Syndrome who have decreased oxytocin-producing neurons [18] and decreased functional *OXTR* [19], together with studies in animals, have underlined the importance of oxytocin and genetics in determining appetite and body mass. For example, in Prader-Willi, both the size of the oxytocin-containing paraventricular nucleus (PVN) and the number of oxytocin neurons within it, are dramatically reduced, which is thought to cause the dramatic hyperphagia seen with the syndrome [18]. Similarly, mice with haploinsufficiency of the *single-minded 1* gene that influences PVN maturation, develop extreme hyperphagia and are susceptible to diet-induced obesity [4]. In the PVN, expression of the synaptotagmin gene results in synaptotagmin-4, a protein that negatively regulates oxytocin release via exocytosis. Increased vesicle binding of synaptotagmin-4 in oxytocin neurons is associated with obesity and mice overexpressing synaptotagmin-4 are obesogenic in contrast to mice deficient in synaptotagmin-4 that are resistant to diet-induced obesity [20].

Oxytocin concentrations have been studied in relation to eating disorders. Low-weight women with anorexia nervosa (AN) have lower serum oxytocin concentrations than healthy controls, and postprandial oxytocin concentrations were associated with ED psychopathology [21]. Additionally, functional magnetic resonance imaging (*f*MRI) hypoactivation of relevant neural reward and homeostatic circuitry was seen in AN and weight-recovered AN, that was associated with plasma concentration oxytocin and disordered eating psychopathology [22, 23].

### OXTR overview

There is evidence to support that sensitivity to oxytocin, which is thought to be mediated by genetic and epigenetic alterations in the OXTR gene, might play a role in criterion eating disorder behaviours [24]. The OXTR itself is a G-protein coupled receptor (GPCR) expressed widely in the mammalian brain. The distribution pattern of OXTR is species-specific and indicates the level of sociability for a species. In solitary species, such as the montane vole, the expression of OXTR increases dramatically during the mating season to facilitate breeding activities—including mate bonding and maternal care--behaviours blocked by OXTR antagonists [25, 26]. The striking cell-specific up- and downregulation of OXTRs is atypical of the GPCR superfamily and also occurs in females during parturition. The *OXTR* is stimulated by oestrogen both peripherally and in central hypothalamic sites with behavioural actions [27–29]; however, the degree to which the *OXTR* is oestrogen sensitive remains unclear [30]. Nonetheless, there are gender differences in oxytocin function and *OXTR* expression [31–41].

In humans, the *OXTR* is located on chromosome 3p25, spanning 17 kb, and containing three introns and four exons [31, 42]. The identified variations of the *OXTR* are SNPs—the most common form of DNA variation—and occur when one nucleotide of a base pair is replaced by another nucleotide. The two most studied *OXTR* SNPs in relation to ED are rs53576 and rs2254298, (see Table 1 for their genotypic distributions across ethnicities), with two possible allelic variants, guanine (G) and adenosine (A). An individual may inherit either the G or the A allele for each one of the SNPs of the *OXTR* from each parent and, thus, an individual would be homozygous if inheriting the same allele from both parents (AA or GG) or heterozygous if different alleles are inherited derived from each parent (AG). There are, therefore, three genotypes for each of these SNP: GG, AG, and AA. Since rs53576 and rs2254298 are located in the non-coding third intron of the *OXTR*, there is no effect in the expression of the OXTR protein.

**Table 1:** Summary of Genotypic Distributions for *OXTR* rs53576 and rs2254298

		Frequencies					
		Allele			Genotype		
rs53576							
Population	Number	A	G	Number	AA	AG	GG
African	1194	0.224	0.776	597	0.057	0.33	0.610
American	524	0.343	0.657	262	0,116	0.453	0.431
Asian	1144	0.684	0.316	662	0.472	0.423	0.105
European	1990	0.361	0.639	995	0.132	0.459	0.409
rs2254298							
African	1112	0.238	0.762	561	0.053	0.370	0.577
American	524	0.240	0.760	262	0.083	0.315	0.602
Asian	968	0.322	0.678	484	0.101	0.441	0458
European	2318	0.107	0.893	1159	0.016	0.182	0.802

### OXTR gene in mental Health Research

Variations in the *OXTR* gene were first linked to human mental health, specifically in autism research, that extended the work demonstrating the links between the *OXTR* and sociability in voles. The hypothesis that variation in the *OXTR* was associated with deficits in social behaviour was substantiated by a study linking rs53576 and rs2254298 to autism [43]. Despite inconsistent findings from subsequent autism and *OXTR* studies, research into the broader hypothesis that *OXTR* polymorphisms are associated with social dysfunction has increased and has been extended to explain other psychopathology, such as depression, schizophrenia and ED, as well as general social functioning [44–47].

### Genetic risk factor identification strategies

Broadly, there are two approaches for identifying genetic risk factors associated to a specific phenotype. The first is the candidate-gene approach, which investigates pre-specified gene variations in relation to a specific phenotype. This hypothesis-driven approach differs from data-driven genome-wide association studies (GWAS) and quantitative trait locus mapping that analyse the DNA sequence of the entire genome to identify genetic risk factors for phenotypes, usually diseases that are common in the population. Research into the *OXTR* and ED has so far pursued only the candidate-gene approach. In addition to common genetic variation, rare gene mutation may identify critical mechanisms in pathology.

In addition to genetic variation, changes in the activity of OXTR may be related to other non-genetic regulatory mechanisms of transcription, such as DNA methylation—an epigenetic mechanism involving the transfer of a methyl group onto the C5 position of the cytosine to form 5-methylcytosine. Epigenomic changes might alter DNA accessibility thereby regulating patterns of gene expression [48]. Consistent with an emerging field of inquiry, functional studies have shown that differential methylation of a CpG island in the *OXTR* promoter have been reported in relation to disorders characterized by impairments in social cognition, including Autistic Spectrum Disorder, eating behaviour, such as anorexia nervosa, and problems with facial and emotional recognition [49–52]. However, taken together, these findings are in pressing need of greater phenotypic precision and methodological improvements including longitudinal studies with multiple time-points, validation in larger and more homogeneous samples, and measures of DNA methylation status of other genes within the same functional circuitry, such as the oxytocin transporter gene, *CD38* [53].

### Aims and objectives

The aim of this critical review is to explore research into *OXTR* rs53576 and rs2254298 SNPs and their role in clinical and non-clinical samples with, or displaying behaviours characteristic of, AN, BN or binge-eating disorder.

## Methods

### Inclusion and exclusion criteria

Research was included in this review if it was written in English, peer-reviewed and explored rs53576 or rs2254298 and ED behaviours together (see Table 2 for a summary of papers included and their main findings). Both clinical and nonclinical populations were included, and no date restrictions were imposed. Research exploring other *OXTR* SNPs, the oxytocin gene itself, oxytocin secretion, and oxytocin administration were excluded from this review. Studies examining the *OXTR* gene and parturition have also been excluded, given the upregulation of oxytocin in the third trimester. Research that relies on salivary or plasma oxytocin concentrations has also been omitted as the validity of these measurements is not decided.

**Table 2:** Population Characteristics and Key Findings in Studies of *OXTR* rs53576 or rs2254298 and ED behaviour

Review Subsection	Authors	Sample	Summary of Study Findings - rs53576	Summary of Study Findings - rs225498
*OXTR* Variants and ED	Connelly et al. [54]	Community sample of females	GG genotype more likely to have engaged in vomiting or laxative use as weight loss strategy	
	Micali et al. [55]	Community sample of females	A allele negatively correlated to binging/purging behaviours, GG genotype was at increased risk of engaging in binging and purging	A allele carriers at increased odds of restrictive eating/purging A allele carriers who had experienced poor maternal care at increased odds of binging purging
	Kim et al. [56]	Females (age range not specified) with AN, BN and HC	G allele positively associated with BN, stronger association observed in those with AG genotype	
	Acevedo et al. [57]	Adult females with AN, BN and HC	Positive association between A allele carriers of either/both SNPs and severity of ED symptomatology In those with previous AN, A allele carrier status increased severity of cognitions and eating behaviours	
	Davis et al. [58]	Community sample of adults	No significant effects for either SNP	
*OXTR* and Brain Structure/Function	Sala et al. [59]	Adult females with, or recovered from, AN		Reduced activation of brain areas underpinning social salience and reflection observed in A allele carriers Greater negative connectivity observed in A allele carriers

### Search strategy

Papers for inclusion were identified by searching the Single Nucleotide Polymorphism Database (dbSNP) of Nucleotide Sequence Variation [60] for studies using the rs53576 [*Homo sapiens*] and rs2254298 [*Homo sapiens*] classification, which returned 184 and 109 publications, respectively. These publications were searched for studies that mentioned eating behaviour or eating disorders, leaving only six studies. The GWAS Catalog [61] was also searched for rs53576 and rs2254298, and neither SNP was found. In addition, PubMed and Google Scholar were searched for peer-reviewed articles using the terms rs53576 OR rs2254298 OR oxytocin receptor OR OXTR, AND eating disorder OR anorexia nervosa OR bulimia nervosa OR binge/binging, but no additional papers were found.

### *OTXR* polymorphisms and eating disorders

Only six studies have addressed the association between *OXTR* SNPs and ED behaviours: two in clinical cohorts and two using ED pathology in the general population. Despite this paucity of research, the emerging results are broadly consistent.

A large-scale hypothesis-free investigation of pregnant women from the Avon Longitudinal Study of Parents and Children (ALSPAC) cohort found that participants with the *OXTR* GG genotype in the rs53576 were more likely to have self-induced vomiting or used laxatives for weight loss [54]. However, results should be taken with caution as these were the only significant findings among 22 analyses conducted on the data and, therefore, may be due to chance alone.

In a community sample comprised of 3698 women recruited as part of the ALSPAC, Micali et al. investigated the relationship between the *OXTR* SNPs rs53576 and rs2254298, maternal care and eating behaviours [55]. The study concluded that regarding rs53576 variant, the A allele negatively correlated with binging or purging behaviours and that, conversely, women who were G homozygotes had increased odds of binging or purging behaviours (*p* = .01) The SNP rs2254298 was most strongly associated with restrictive eating behaviour, in particular the A allele, with A-allele carriers and homozygotes having increased odds of engaging in restrictive eating and purging behaviours (*p* < .0001 and *p* = .03, respectively). Poor maternal care was independently positively associated with binging and purging but only interacted significantly with the rs2254298 A allele, carriers of which were four times more likely to engage in binging/purging (*p* = .03). The study’s strengths lie in its large sample size and longitudinal design, which allowed for evaluation of eating behaviours over time; however, the authors recognise that given the low frequency of certain eating behaviours, the sample may not have been sufficiently powered to identify small associations.

In a clinical sample of 90 women with bulimia nervosa (BN) examining six *OXTR* SNPs, which included rs53576 and rs2254298, the G allele of rs53576 was positively correlated with BN [56]. However, the authors acknowledge that results were not corrected for multiple testing and, had the alpha level been lowered, the significant association between genotype and BN would have disappeared. In addition, the heterozygous genotype (AG) was more strongly associated with BN than the homozygous (GG), creating interpretative difficulties in asserting that the G allele confers risk for BN. No further associations were found among genotype distributions or allele frequencies for the other *OXTR* gene SNPs and AN or BN.

A study investigating the link between ED symptomatology and five *OXTR* SNPs in a sample of 124 females with and without ED found a positive correlation between rs53576 and rs2254298 in the A-allele carriers and the severity of ED symptomatology [57]. Moreover, for individuals with a previous history of AN, carrying at least one copy of the A allele for both the rs53576 and rs2254298 SNPs, was associated with increased ED symptom severity in relation to both cognition and eating behaviours. This finding also suggests that rs53576-rs2254298 GG/GG haplotypes may confer some protective effect as evidenced by lower reported anxiety and preoccupation with body and food in both healthy controls and those with a previous history of AN and in weight recovery.

A further exploratory study in a community sample of 460 adults examined the association between overeating and seven *OXTR* SNPs, including rs53576 and rs2254298, reported no significant effects with respect to either SNP [58]. However, linkage disequilibrium analysis revealed that within a four SNP haplotype, the G-T-A-G haplotype formed of the rs237885, rs2268493, rs2268494 and rs2254298 respectively, was associated with higher food preference scores. Food preference scores were a measure of preference of high fat and/or sugar foods—those characteristic of a binge—thus it may be inferred that the G allele of rs225498 somewhat contributes to the risk of binging behaviours.

### OXTR polymorphisms and brain structure/function

Only one brain imaging study has so far examined the *OXTR* rs53576 or rs2254298 and ED (see Table 2). Using *f*MRI, Sala et al. evaluated neural responses to a social attribution task in adult women with, or recovered from, AN [60]. In line with the finding that rs2254298 AG or AA genotypes were associated with restrictive eating behaviours [55], females with AN had reduced activation of brain areas subserving social salience and reflection during a social attribution task [59]. Specifically, reduced activation of principal components of the default mode network (medial prefrontal cortex, posterior cingulate cortex, and precuneus) that underpin introspective decision-making and thinking about self or others was seen in rs2254298 A carriers relative to rs2254298 G homozygotes. In addition, negative connectivity among the posterior cingulate cortex, the occipital lobe and the cerebellum was greater in rs2254298 A heterozygotes than in rs2254298 GG homozygotes, meaning activation patterns in areas associated with bottom-up attention, vision and voluntary body movement were negatively correlated. However, data from this study may not be reliable due to its small sample size and unequal group numbers.

### Gene-environment interactions in ED

If an increased risk for a phenotype is conferred by an environmental factor, then a gene by environment (G x E) interaction applies. Only Micali et al. in their abovementioned study also addressed G x E interactions in relation to a disordered eating phenotype [55]. They found that, although the rs2254298 AA or AG genotypes were associated with restrictive eating behaviour, when poor maternal care was present, rs2254298 AA or AG genotypes had a four-fold increase in the odds of binge eating and purging (*p* = .03) [55].

A number of environmental factors that relate indirectly to ED or allied phenotypes, such as obesity, have also been explored via G x E studies of the *OXTR* rs53576 and rs2254298 SNPs. A longitudinal study of 192 children examined *OXTR* rs53576 and its interaction with socioeconomic status to predict the risk of childhood obesity [61]. Authors found that the A allele of rs53567 predicted body mass when socioeconomic status was accounted for [62]. This finding reinforces oxytocin’s role in factors that might drive childhood obesity such as metabolic regulation, appetite, and reward-driven eating processes and underlines the importance of studying genetic predispositions to health outcomes. Promising interactions between childhood adversity and rs53576 on later development of psychopathology have been described in three separate G x E studies [63–65]. Although these interactions were not replicated in a later study [66], the neurostructural and functional underpinnings of this *OXTR* rs53576 × childhood adversity interaction have been investigated using *f*MRI and found to map to the ventral striatum in the reward area of the brain [67]. In GG homozygotes, ventral striatum grey matter volume negatively correlated with the degree of childhood maltreatment, but this detrimental outcome was not seen in A-allele carriers [56]. Interestingly, a review discussing early-life caregiver deprivation and the later development of depression proposed a role for the ventral striatum [68]. Given the role of oxytocin in childhood bonding [24, 69–81], these findings are, perhaps, unsurprising. However, attachment and early years’ experiences are also established risk factors for the development of ED [82–86], so further research to investigate the impact of the interaction of *OXTR* polymorphisms and childhood environment on eating behaviours would be useful.

### Limitations

There are a number of difficulties regarding reliability, interpretation, and validity of candidate-gene association studies. Results are often not replicated, and it is not known how often failure to replicate has resulted in failure to publish. In addition, small sample sizes in both original and replication studies create problems of lack of power highlighted as particular problem for both candidate-gene research and neuroscience [87].

There are specific issues pertaining to candidate-gene research using *OXTR* SNPs. Few studies compare data between women and men, despite evidence of sex-specific effects [88–91] and few studies account for ethnicity, although the minor allele for rs53576 is different in Caucasian and Asian groups. In addition, the same outcomes are rarely measured, making the comparison of results difficult.

## Conclusion

Understanding an aetiologic role for oxytocin in the development of ED is important, not only for researchers, but also for clinicians and patients, with potential new treatment avenues becoming an option for the latter group. However, despite the association of rs53576 and rs2254298 with eating patterns in a non-clinical population study, so far, these SNPs have not been identified in genome-wide association studies examining eating behaviours or obesity. Individual *OXTR* SNPs are unlikely in themselves to explain complex eating disorders but may form part of a haplotype with other *OXTR* SNPs to affect the expression of and/or effectiveness of the OXTR. A growing body of G x E work is indicating that rs53576G homozygosity becomes disadvantageous for later mental health under early adverse conditions but further research to extend these findings to eating pathology is needed. Eating disorders are not genetic monoliths but complex behaviours arising from multigenetic-environment interactions over time and the GWAS approach would benefit this area of knowledge.

## Data Availability

Not applicable.

## Abbreviations

ED: Eating disorders
*f*MRI: Functional magnetic resonance imaging
ICV: Intracerebroventricular
OXTR: Oxytocin receptor
*OXTR*: Oxytocin receptor gene
PVN: Paraventricular nucleus
SNP: Single nucleotide polymorphism
